# Economic evaluation of tele-resuscitation intervention on emergency department pediatric visits in the Niagara Region, Canada a pilot study

**DOI:** 10.3389/frhs.2023.1105635

**Published:** 2023-06-05

**Authors:** A. Pace, B. E. Faught, M. Law, L. Mateus, M. Roy, C. Sulowski, A. Khowaja

**Affiliations:** ^1^ Department of Health Sciences, Brock University, St. Catharines, ON, Canada; ^2^ Niagara Health, Niagara Region, ON, Canada; ^3^ Pediatric Department, McMaster Children’s Hospital, Hamilton, ON, Canada

**Keywords:** cost-effectiveness, pediatric resuscitation, telemedecine, economic analysis, societal perspective

## Abstract

**Introduction:**

The use of telemedicine in critical care is emerging, however, there is a paucity of information surrounding the costs relative to health gains in the pediatric population. This study aimed to estimate the cost-effectiveness of a pediatric tele-resuscitation (Peds-TECH) intervention compared to the usual care in five community hospital emergency departments (EDs). Using a decision tree analysis approach with secondary retrospective data from a 3-year time period, this cost-effectiveness analysis was completed.

**Methods:**

A mixed methods quasi-experimental design was embedded in the economic evaluation of Peds-TECH intervention. Patients aged <18 years triaged as Canadian Triage and Acuity Scale 1 or 2 at EDs were eligible to receive the intervention. Qualitative interviews were conducted with parents/caregivers to explore the out-of-pocket (OOP) expenses. Patient-level health resource utilization was extracted from Niagara Health databases. The Peds-TECH budget calculated one-time technology and operational costs per patient. Base-case analyses determined the incremental cost per year of life lost (YLL) averted, and additional sensitivity analysis confirmed the robustness of the results.

**Results:**

Odds ratio for mortality among cases was 0.498 (95% CI: 0.173, 1.43). The average cost of a patient receiving the Peds-TECH intervention was $2,032.73 compared to $317.45 in usual care. In total, 54 patients received the Peds-TECH intervention. Fewer children died in the intervention group resulting in 4.71 YLL. The probabilistic analysis revealed an incremental cost-effectiveness ratio of $64.61 per YLL averted.

**Conclusion:**

Peds-TECH appears to be a cost-effective intervention for resuscitating infants/children in hospital emergency departments.

## Introduction

Inadequate resources and all-day unavailability of trained health care personnel in the smaller or remote emergency departments (EDs) is a significant concern in Canada (1). Infants and children requiring critical care and/or resuscitation are often faced with issues related to staff shortages and delayed care resulting in severe consequences (2). Additionally, there are considerable financial implications associated with intensive care and longer hospital stay for parents/caregivers of children and the health care system overall. Telemedicine (TM) has been growing rapidly to address these issues surrounding the lack of available specialists or inadequate access to resources. This is especially true among pediatric patients as research has deemed TM to have clinical effectiveness in pediatric medicine where specialists are unavailable (3, 4). When assessing cost-effectiveness, there is conflicting evidence regarding TM. Although there is a plethora of health economics literature favouring TM over the previous standard of care; these studies often do not include a sufficiently broad perspective to fully encompass all the necessary costs associated with TM (5–9). This lack of perspective results in skewed outcomes regarding the true cost-effectiveness of many TM programs. This serves as the impetus for the current study which utilized a broad societal frame of reference.

The goal of Peds-TECH is to enhance pediatric outcomes for infants and children who present to Niagara Health (NH) hospitals' emergency departments. This intervention is used only in the most severe cases where receiving physicians/nurses believe they require assistance from pediatric specialists at the tertiary hospital. The Peds-TECH technology enables clinical experts to communicate with the care team via videoconferencing, providing guidance on best practices for resuscitation situation. Due to the severe nature of the call for Peds-TECH, clinical judgment is required to activate the intervention in a timely manner. The intervention's primary goal is to stabilize the patient before transfer to a tertiary hospital. This study included EDs of five community hospitals in the Niagara Region, which served as the entry point for patients. Following entry, pediatric patients were classified as CTAS-1 or CTAS-2. CTAS-1 refers to patients with the most urgent and life-threatening conditions who require immediate attention, while CTAS-2 refers to patients with conditions that are potentially life-threatening and require rapid assessment and treatment**.** Therefore, if patients are <18 years, triaged as CTAS 1 or 2, and clinical judgement is used to trigger the call for Peds-TECH, patients are eligible for the intervention. At the time of this study, 54 patients had been treated using this technology. At present, there is a knowledge gap regarding the economic impact of the Peds-TECH intervention. As well, additional hospitals in surrounding areas have expressed interest in the Peds-TECH intervention. Therefore, it was crucial to evaluate the costs and cost-effectiveness of the intervention to inform decisions on resource allocation for further spread in the Niagara region and the province of Ontario at large. This study aimed to assess societal costs and cost-effectiveness of the intervention including (i) the out-of-pocket (OOP) costs and time/productivity gains/losses for parents of children seeking care at the community hospital EDs, (ii) costs associated with the health resource utilization (HRU) at the health facility, and (iii) the Peds–TECH technology and program implementation costs. The time horizon of our study was three years, enabling us to capture short-term costs and health outcomes associated with the Peds-TECH intervention and provide evidence to inform resource allocation decisions for its further implementation in the Niagara Region and beyond (10).

## Methods

A mixed methods quasi-experimental design was embedded in the economic evaluation of Peds-TECH intervention compared with usual emergency care (control group). This study was conducted over three years (December 2018–2021) in five community hospital EDs in the Niagara Region, Canada. This study followed the Canadian Agency for Drugs and Technologies in Health guidelines for conducting an economic evaluation of health technologies using a societal perspective (11). We also followed the Consolidated Health Economic Evaluation Reporting Standards for health economic reporting (12).

### Participant eligibility criteria

According to Canadian triage protocols, Canadian Triage and Acuity Scale (CTAS)-1 patients were classified as those requiring resuscitation, while CTAS-2 were considered emergent cases (13). All infants and children aged <18 years triaged as CTAS-1 or 2 were eligible to receive Peds-TECH intervention. Receiving physicians in ED triaged and triggered the call for Peds-TECH intervention based on clinical presentation and severity. Children of all sexes <18 years of age consisting of CTAS-1 and CTAS-2 receiving the Peds-TECH intervention were classified as cases. Control patients were matched using a one-to-one simple matching design, where each case (patient treated with the Peds-TECH intervention) was matched with one control patient based on hospital location and CTAS designation. Additionally, we matched on age (within 1 month) and gender to further control for potential confounding factors. These variables are important as they can impact the acuity, severity and level of care of a patient's condition. Matching on these four key variables provided adequate control for potential confounding variables (14). The descriptive statistics of cases and controls are reported in Table 1.

**Table 1:** Descriptive statistics of cases and matched controls.

Variables	Cases, *N* = 54		Matched Control, *N* = 54	
	CTAS-1 *n* (%)	CTAS-2 *n* (%)	CTAS-1 *n* (%)	CTAS-2 *n* (%)
Pediatric Patients	41 (75.9)	13 (24.1)	41 (75.9)	13 (24.1)
**Age categories**				
<1 month	5 (12.1)	0	6 (14.6)	1 (7.7)
1–12 months	4 (9.8)	3 (23.1)	4 (9.8)	2 (15.4)
13 Months to ≤5 Years	14 (34.1)	6 (46.2)	14 (34.1)	3 (23.1)
>5 Years to 18 Years	18 (43.9)	4 (30.8)	17 (41.5)	7 (53.8)
Average Age in Years (SD)	5.24 (5.16)	3.69 (3.55)	6.50 (6.51)	6.58 (5.70)
*p*-value for age	*p* = 0.044			
**Sites**				
Fort Erie ED	2 (4.9)	1 (7.7)	2 (4.9)	1 (7.7)
Niagara Falls ED	12 (29.3)	4 (30.8)	12 (29.3)	4 (30.8)
St. Catharines ED	11 (26.8)	5 (38.5)	11 (26.8)	5 (38.5)
Welland ED	16 (39)	3(23.1)	16(39)	3(23.1)

### Cost calculations & statistical analysis

Qualitative semi-structured interviews were conducted with five parents of patients undergoing Peds-TECH intervention to identify OOP expenses, and productivity losses for families whose child visited the ED or were transferred to a tertiary hospital (15). During the interviews, participants were asked about their hourly wage and the number of days missed from work due to their child's hospitalization. This allowed us to calculate an average cost related to productivity loss for families whose child visited the ED or were transferred to a tertiary hospital, which was included in the economic model. Study participants were recruited using convenience sampling based on their availability for a 40–60 min virtual interview. The interviews were conducted by two graduate student research assistants and written notes were taken during each interview. The audio-visual recordings were undertaken for each interview and the transcripts were auto generated using the teleconferencing application. Interviews were analyzed using both deductive content and inductive thematic analyses (16). Findings from the interviews informed the economic model design and generated OOP cost parameters for the cost-effectiveness analysis.

We extracted patient-level health resource utilization data from secondary clinical databases at NH. Descriptive analysis was undertaken to calculate frequency, proportions, mean and standard deviation of participant's characteristics and HRU. A *t*-test was applied to compare the mean age, costs, and YLL between intervention and control with the aim of determining the statistical differences and *p*-values to support the interpretation of the results, which are found in Tables 1, 2. Additionally, we calculated the Odds ratio (OR) and subsequent 95% CI resulting from mortality between the two groups. This was completed through *χ*^2^ analysis, where a 2 × 2 contingency table was created between those who died or survived. The OR was used to estimate the probability of mortality in the intervention group relative to the control group. All descriptive and statistical analyses were undertaken in the SPSS package software version 28.0.

**Table 2:** Base case analysis results for Peds-TECH intervention compared to standard of care.

Base Case Results	Cases, *N* = 54	Matched Control, *N* = 54	*p*-value
Costs	$2,032.73	$317.45	<0.001
YLL	4.71	31.26	<0.001
ICER	$64.61 per YLL averted		

A costing proforma was created to determine the financial costs associated with Peds-TECH program implementation. The sum of these costs was divided by the number of cases, to determine a cost per case. Additionally, the public-payer costs to the health care system were calculated as HRU per patient. We extracted clinical information from secondary clinical databases at NH to determine the frequency of HRU, including time spent in ED, services received, and emergency transfers. The standard cost of an ED visit reported by the Canadian Institute for Health Information (CIHI) was multiplied by HRU to calculate the ED cost per patient (17). All costs were represented in Canadian currency as of 2022.

### Decision tree analysis

**Figure 1:**
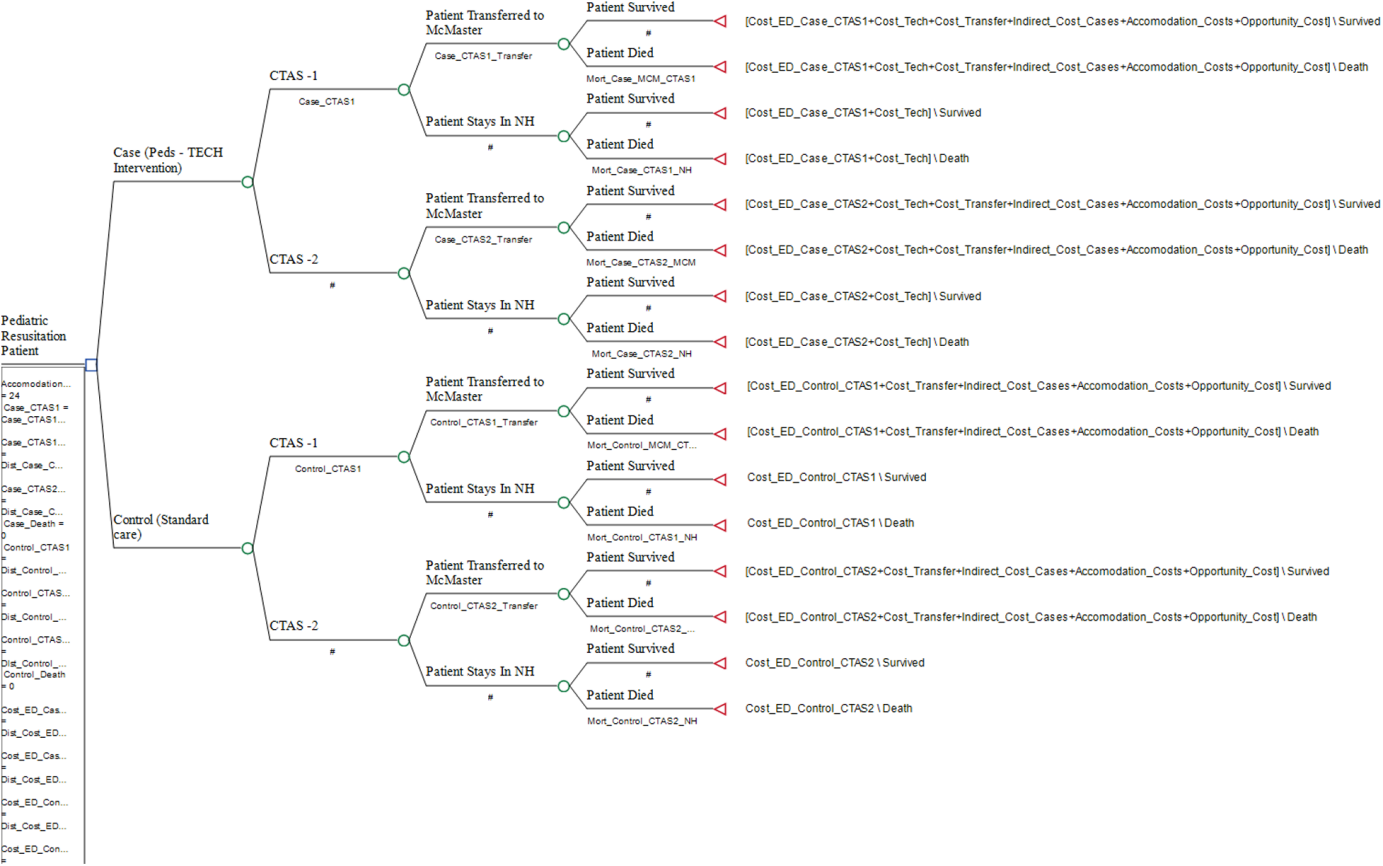
Decision tree model of peds-TECH intervention.

A decision-tree analytic model (DAM) was applied to capture all necessary pathways in the clinical trajectory of care, transfers, and the survival/mortality outcome for children undergoing the Peds-TECH intervention and was conducted using TreeAge 2022 software. The primary clinical endpoint for children was survival/mortality, which was translated into years of life lost (YLL), based on the average life expectancy seen in Canada. The decision tree with respective branches is shown in Figure 1. The findings from the cost-effectiveness analysis are reported as the incremental cost-effectiveness ratios (ICERs) per YLL averted. Finally, we conducted sensitivity analyses to control for uncertainties associated with cost and outcome parameters, which included one-way sensitivity analysis and probabilistic sensitivity analysis using 10,000 runs through a Monte Carlo simulation (18). In the univariate sensitivity analysis, we varied the costs of Peds-TECH program implementation, the cost of an ED visit, the frequency of healthcare resource utilization, and the probability of survival/mortality outcomes by 20% to assess the impact of these parameters on the cost-effectiveness results. Performing univariate sensitivity analyses allowed us to assess the robustness of the cost-effectiveness results to changes in key input parameters and identify which parameters had the greatest impact on the cost-effectiveness results. Allowing us to explore the range of values over which the intervention remained cost-effective.

## Results

### OOP costs and productivity losses

Qualitative interviews with five parents of children treated with Peds-TECH highlighted average OOP expenses pertinent to parking/travel to and from the hospital. The largest category in terms of the overall cost ($435/patient) was parking/travel costs. Expenses related to food/meals were $80/patient and productivity losses based on time from missed work, were determined to be $160/patient. These costs were applied in our decision tree model to all patients who were transferred to a tertiary hospital in both cases and controls.

### Costs of health resource utilization

Data from 108 children (cases *n* = 54; matched controls *n* = 54) diagnosed as either CTAS-1 or CTAS–2 were included in the economic analysis. Our cases consisted of 41 CTAS-1 and 13 CTAS-2. The average age for all cases was 4.86 years and 6.52 years for controls. There was a significantly higher rate of transfer in cases (77.8%) compared to controls (16.6%) (*p* < 0.01). Therefore, higher costs were identified for both transfer and OOP time and travel costs related to transfers amongst cases. HRU costs were also included in the economic model such as ED costs per case/control, technology costs or costs related to transfer. All HRU costs and their description are reported in Table 4.

### Mortality outcome

The cases had seven total deaths, with a mean age among those who died at 5.48 years. Similarly, the control group had 12 deaths with a mean age of 5.48 years. There were no reported deaths among those who were designated as CTAS-2 in either group. There was also no mortality among those transferred in either group. Table 1 shows the characteristics of pediatric patients who received the Peds-TECH intervention and the matched control group, including their CTAS designation, age, and hospital location. The odds ratio for mortality among cases was 0.498 (95% CI: 0.173, 1.43 *p* = 0.23) compared to controls, indicating some degree of uncertainty associated with mortality. Although the *p*-value for mortality was not significant the OR for mortality indicates a potential protective effect of the Peds-TECH intervention. However, further research with a larger sample size is needed to confirm this finding.

### Cost-effectiveness of Peds-TECH

**Figure 2:**
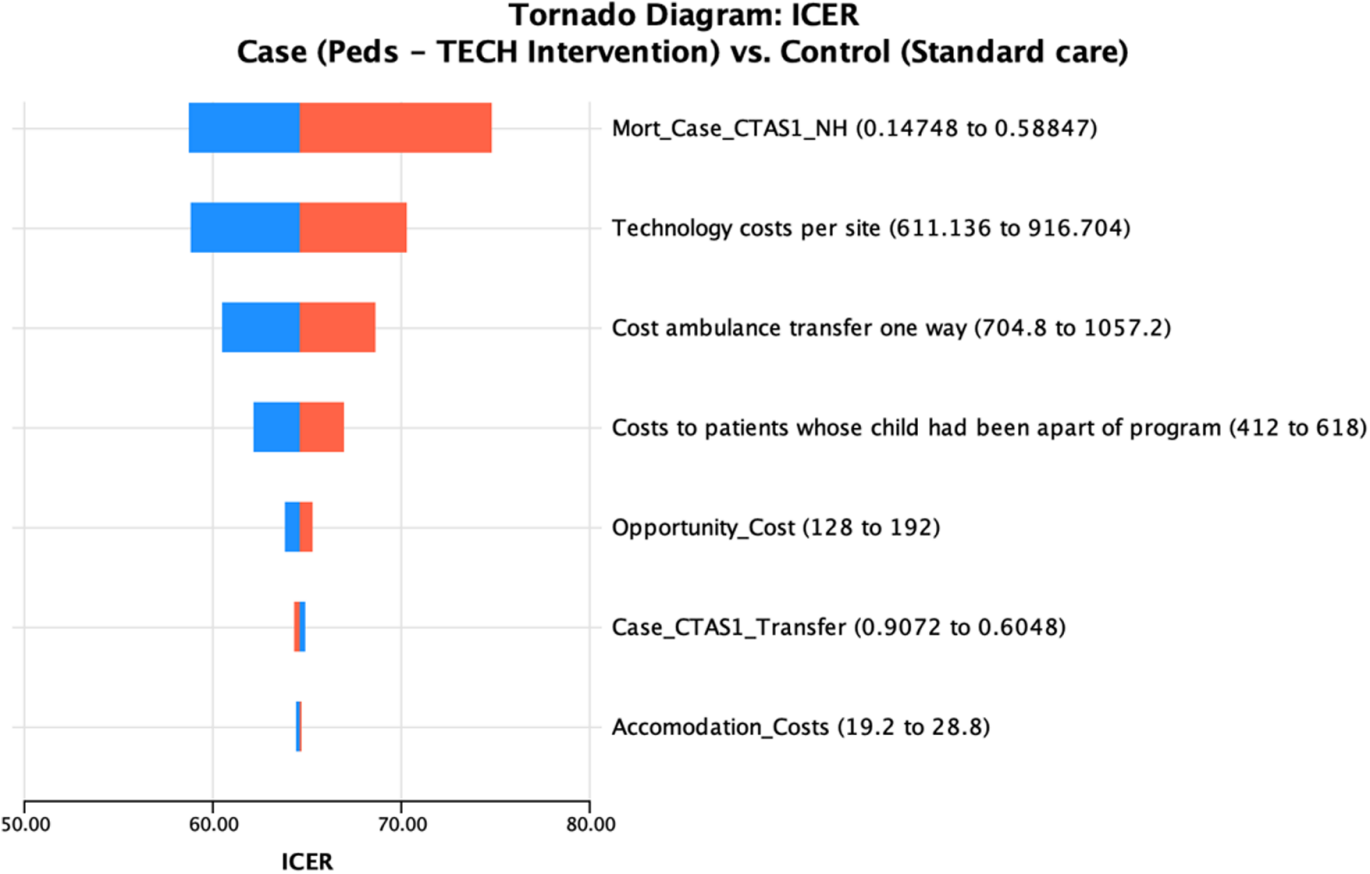
Tornado diagram results from 1–way sensitivity analysis of sensitive variables from decision tree model. The red bars represent the ICER when the high value is included in the model, the blue bars represent the ICER when the low value is included.

The base case analysis results are presented in Table 2, the mean cost among cases was $2,032.73 and the mean effect was 4.71 YLL. For controls, the mean cost was $317.45, and the mean effect was 31.26 YLL. In the incremental analysis, we estimated an ICER of $64.61 per YLL averted. This ICER value represents the cost for one additional life-year averted in the intervention group compared to the control. One-way sensitivity analysis that reflected the change in ICER are illustrated in a tornado diagram (Figure 2). Cost parameter values and their description is shown in Table 3. Additionally, the breakdown of indirect and direct costs for the intervention and standard of care can be seen in Table 4.

**Table 3:** Cost parameter values, with a brief description of what they represent, along with their source.

Parameter	Value (CAD)	Description	Source
ED Cost per CTAS-1 Case	$40.52	Average cost of an ED visit of someone CTAS-1, treated with Peds- TECH intervention.	Calculated, (CIHI, 2020)
ED Cost per CTAS-2 Case	$38.11	Average cost of an ED visit of someone CTAS-2, treated with Peds-TECH intervention.	Calculated, (CIHI, 2020)
ED Cost per CTAS-1 Control	$53.76	Average cost of an ED visit of someone CTAS-1, not treated with Peds—TECH.	Calculated, (CIHI, 2020)
ED Cost per CTAS-2 Control	$55.88	Average cost of an ED visit of someone CTAS-2, not treated with Peds—TECH.	Calculated, (CIHI, 2020)
Cost of Technology per case (intervention only)	$763.92	Average cost of the Peds-TECH intervention, per case. Applied to cases only.	Project Financials
Cost of Transfer per child	$881	Average cost of a one-way ambulance transfer to tertiary hospital. Applied to both cases and controls who were transferred.	(ICES, 2009)
OOP time and travel costs	$515	Average OOP costs calculated from qualitative interviews with parents of patients whose children were treated with Peds—TECH. Applied to both cases and controls who were transferred to tertiary hospital.	Estimated, Qualitative interviews
Accommodation Costs	$24	Average costs related to accommodation calculated from qualitative interviews. Applied to both cases and controls who were transferred to tertiary hospital.	Estimated, Qualitative interviews
Productivity Losses	$160	Average costs related to missed work resulting from child being treated with Peds-TECH, calculated from qualitative interviews. Applied to both cases and controls who were transferred to tertiary hospital.	Estimated, Qualitative interviews

**Table 4:** Breakdown of costing for intervention and control.

Costs	Cases		Controls	
Direct Costs	CTAS-1	CTAS-2	CTAS-1	CTAS-2
HRU	$40.52	$38.11	$53.76	$55.88
Technology Costs	$763.92	$763.92	–	–
Transfer Costs (If applicable)	$881	$881	$881	$881
**OOP Costs** (Applied only to those who were transferred to a tertiary hospital)				
Transport	$435	$435	$435	$435
Food	$80	$80	$80	$80
Accommodation	$24	$24	$24	$24
Productivity Losses	$160	$160	$160	$160
Total if transferred	$2,384.44	$2,332.82	$1,633.76	$1,635.88
Total no transfer	$804.44	$802.03	$53.76	$55.88

**Figure 3:**
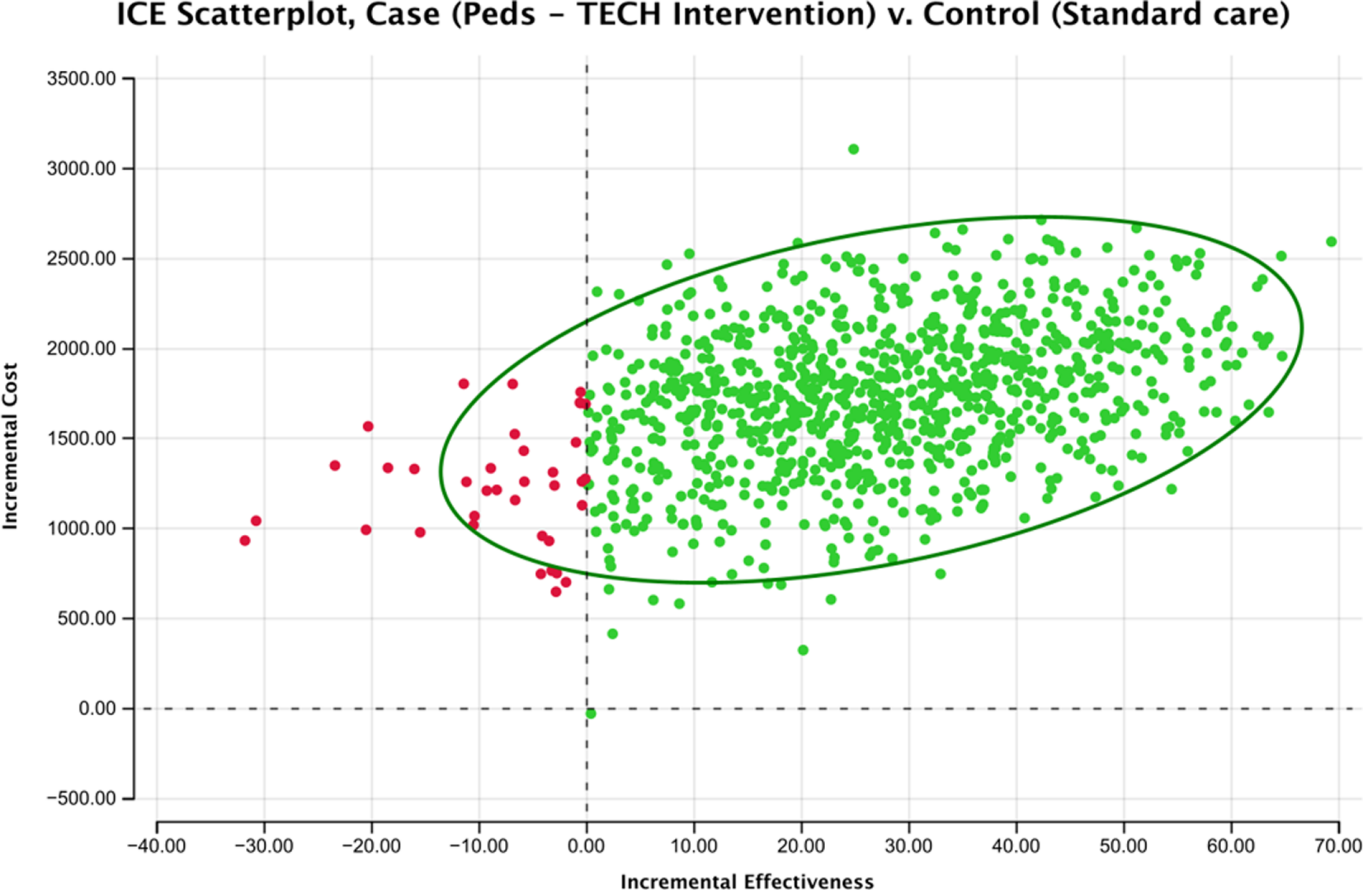
ICE scatterplot results from PSA. Most points fall in the NE quadrant, showing the intervention is more costly, and more effective.

**Figure 4:**
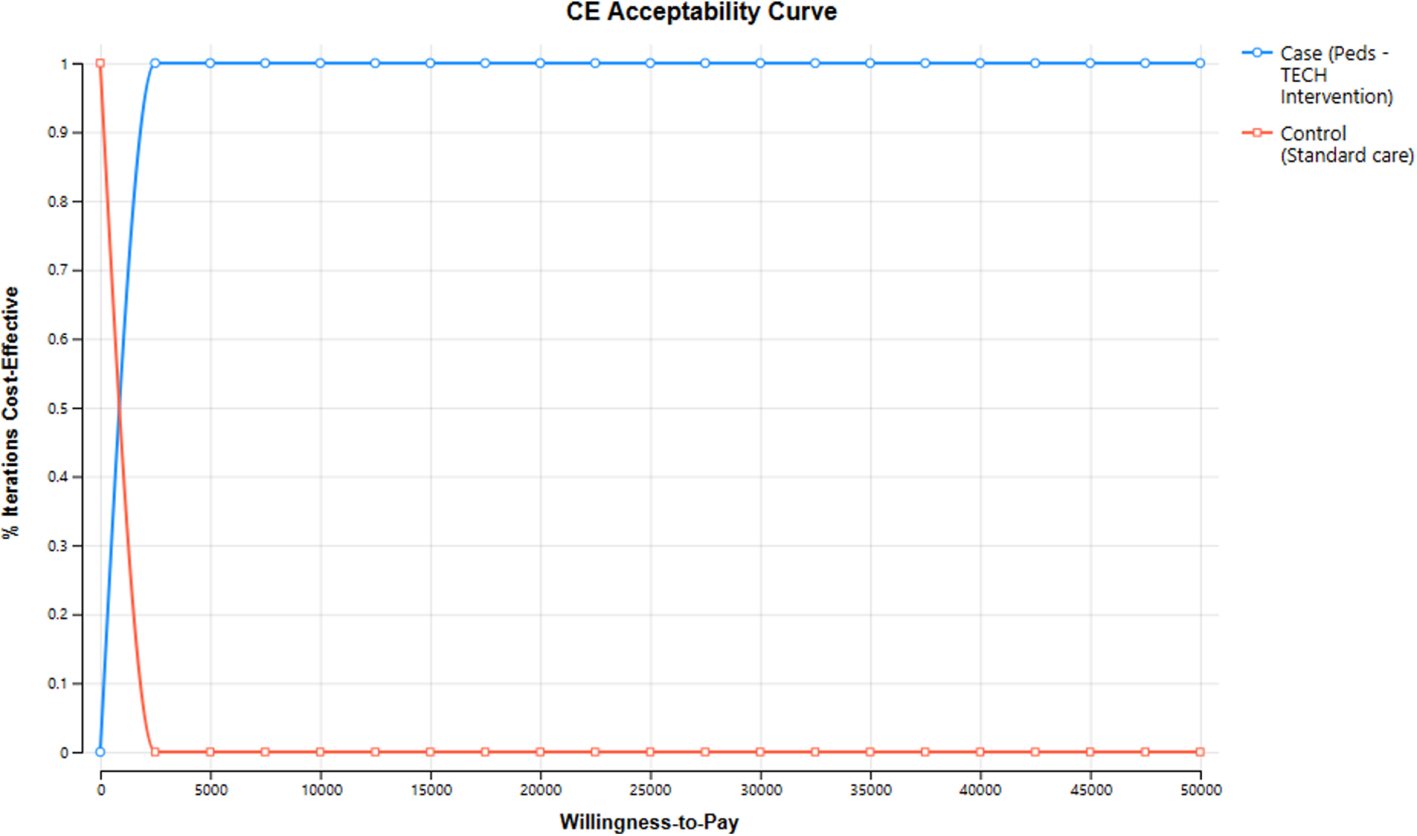
Cost-effectiveness acceptability curve results from PSA, at $5,000 willingness-to-pay there is 96.1% certainty of cost-effectiveness.

The results of the probabilistic sensitivity analysis (PSA) demonstrate the impact of parameter uncertainty on the incremental cost-effectiveness ratio (ICER), which was estimated to be $64.91 per YLL averted (95% credible interval of −138.72–489.75), highlighting the range of potential ICER values that could arise due to variations in the model inputs.. Therefore, even after 10,000 random samples with a variance range of 20%; the intervention retains cost-effectiveness. This is reflected in Figure 3, where resulting incremental cost-effectiveness scatterplot illustrates that most of the points fell in the northeast quadrant. This indicates that the intervention was both more costly and more effective than the usual care. The scatterplot also indicated some uncertainty in the results, with some simulations showing the intervention to be less effective or more costly than the usual care. However, the overall trend in the scatterplot suggests that the intervention was a cost-effective option for managing pediatric emergencies in the ED setting. Each variable that was included in the PSA and information on their parameter, uncertainty range, and distribution type are reported in Table 5. As well, the probabilistic analysis indicated a 96.1% probability that Peds-TECH is cost-effective under a $5,000 WTP threshold, which is much lower than the standard $50,000 WTP threshold (Figure 4).

**Table 5:** Parameters included in PSA, the range of uncertainty and type of distribution included.

Name Of Parameter	Value	Uncertainty Range	Distribution	Source
ED Cost per CTAS-1 Case	$40.52	8.03–99.17	Gamma	Calculated, (CIHI, 2020)
ED Cost per CTAS-2 Case	$38.11	6.42–97.79	Gamma	Calculated, (CIHI, 2020)
ED Cost per CTAS-1 Control	$53.76	0.132–252.2	Gamma	Calculated, (CIHI, 2020)
ED Cost per CTAS-2 Control	$55.88	1.21–210.32	Gamma	Calculated, (CIHI, 2020)
Cost of Technology per case	$763.92	$494.65–$1,091	Gamma	Project Financials
Cost of Transfer per child	$881	$570.13–$1,258	Gamma	(ICES, 2009)
OOP costs per child	$515	$333.28–$735.63	Gamma	Estimated, Qualitative interviews
Productivity Losses	$160	$128–$192	Gamma	Estimated, Qualitative interviews
Accommodation Costs	$24	$19.2–$28.8	Gamma	Estimated, Qualitative interviews
Proportion of Cases CTAS-1	0.759	0–0.995	Beta	Calculated, NH Data
Proportion of Controls CTAS-1	0.759	0–0.995	Beta	Calculated, NH Data
Proportion of Cases CTAS-1 transferred	0.756	0–0.995	Beta	Calculated, NH Data
Proportion of Cases CTAS-2 transferred	0.846	0–1	Beta	Calculated, NH Data
Proportion Controls CTAS-1 Transferred	0.195	0–1	Beta	Calculated, NH Data
Proportion Controls CTAS-2 Transferred	0.077	0–1	Beta	Calculated, NH Data
Proportion of Deaths Cases	0.332	0–1	Beta	Calculated, NH Data
Proportion of Deaths Controls	0.668	0–1	Beta	Calculated, NH Data

### Ethical consideration

This study received approval from the Institutional Research Ethics Boards. Informed consent was obtained from the parents of patients prior to the interviews.

## Discussion

### Principal findings

This study highlights the cost-effectiveness of the Peds-TECH intervention to enhance pediatric outcomes for infants and children. Applying a societal perspective to evaluating the costs of young children presenting to EDs, we found that each additional life-year saved cost $64.61 (CAD), which could be considered highly cost-effective compared to the standard $50,000 WTP threshold in Canada (8). Notably, the primary driver of this incremental cost came from a higher rate of transfer among cases (78%), leading to overall higher costs of $881 per transfer. This could be attributed to the expertise of remote pediatricians who provided support to the ED staff at community hospitals, leading to more patients being transferred to tertiary care hospitals for specialized care. While this resulted in higher costs for transfer, OOP time and travel costs and indirect costs, such as productivity losses, it is important to note that patients treated with the Peds-TECH intervention spent less overall time in the emergency department, resulting in lower HRU costs related to length of stay (Table 6). Including productivity losses in the analysis provided a more comprehensive understanding of the economic burden that families face due to their child's hospitalization. Overall, using qualitative interviews to estimate productivity losses allowed us to obtain a more accurate and personalized estimate of the indirect costs associated with the child's hospitalization. Additionally, while there was no statistically significant difference in reduction of mortality between the study's two groups, the mortality reduction in the intervention group highlights the role of physicians and/or nurses in EDs to stabilize patients prior to transfer to a tertiary hospital. It is possible that the higher rate of transfers and subsequent treatment by specialists among cases contributed to the lower risk of mortality observed in this group, as compared to the control group. Furthermore, the costs related to the implementation of the Peds-TECH technology ($763.92/patient) was another factor in the higher costs shown among those treated with the intervention. The overall program cost, however, is considered low when discussing telemedicine ED implementation, which has been shown to have total costs range between $50,000–$100,000 on average (19).

**Table 6:** Outcomes for cases and matched controls.

Variables	Cases, *N* = 54		Matched Control, *N* = 54	
	CTAS-1 *n* (%)	CTAS-2 *n* (%)	CTAS-1 *n* (%)	CTAS-2 *n* (%)
Transfer				
Transferred to McMaster	31 (75.6)	11 (84.6)	8 (19.5)	1 (7.7)
ED visit				
Mean length of stay in hours (SD)	3.03 (1.78)	2.85 (1.79)	4.02 (5.27)	4.178 (4.27)
Mortality				
Yes	7 (17.7)	0	12 (29.2)	0
Odds Ratio Mortality (95% CI)	0.5 (0.17, 1.4; *p* = 0.23)	–		

Societal costs of $515 per patient identified in this study are much higher compared to other studies such as Langabeer and colleagues that reported a societal cost average of $270 pre-implementation and $167 post-implementation per patient (20). This can be explained by the large amount of travel for parents of patients treated with Peds-TECH. The average distance to the tertiary hospital was 78 km and the average number of trips made by parents was five. Generally, travel costs are reduced by the implementation of a TM program as seen in the study by Ellis et al. (9). Although the Peds–TECH intervention differentiates as it is initiated when patients are under extreme circumstances. Therefore, the primary goal of health care providers is patient stabilization prior to transfer to pediatric specialists. For this reason, reducing transfers and their related costs is not feasible when assessing the goals of patient stabilization and reduction of mortality.

This economic analysis albeit yielded lower ICERs, there was considerable parameter uncertainty observed in the mortality estimates. We found no statistically significant difference in reduction of mortality between the two groups of the study. Despite the odds ratio for mortality being protective among cases (0.498), the CI intervals (the upper and lower bounds) were wide. This could be primarily due to a lower sample size in our study and the fact that mortality is a rare outcome in high-income country settings. Although the statistical *p*-value for the mortality variable was not favorable, the magnitude (i.e., the effect size) and the direction of the effect indicated positive gains (i.e., life saved). Therefore, the mortality reduction in the intervention relative to the control group highlights the role of physicians and/or nurses in EDs to stabilize patients prior to transfer to a tertiary hospital. Consequently, more patients were treated by specialists upon their arrival, which may have lowered the risk of mortality. Our findings are further corroborated with other studies surrounding outcomes related to TM implementation. Sadaka et al. (21) reported a reduction in ICU mortality during post intervention as well as an overall decreased hospital mortality. Similarly, Breslow et al. (22) found that during their remote ICU period, mortality for their patients was lower and hospital length of stay was shorter. Finally, McCambridge et al. (23) found that overall hospital mortality decreased in their intervention group, compared to the control group. They attributed this to the increased access of specialists and medical staff, provided by the TM program. Overall, the standard of care received by patients treated with the intervention compare favourably to those not treated with the intervention. Although there is a minimal increase in costs, the outcomes of decreased mortality, increased access to specialists, and shorter length of stay make treatment with the intervention more effective. Additionally, the ability of the intervention to help with the stabilization of patients prior to transfer is an important outcome that has potential to reduce mortality in the future.

### Strengths and limitations

Aside from being a novel advancement in health economics research relating to TM; this study was the first to assess the cost-effectiveness of the Peds-TECH intervention. This research also provided an understanding of the OOP parental costs associated with emergency pediatric patients, which were largely associated with travel to tertiary hospitals. Our findings coincide with the literature surrounding the implementation of TM programs and improved clinical outcomes for patients. This finding is crucial as the use of the technology has the potential to lower mortality in the future. One of the limitations of this study was the small number of parent interviews and the inability to interview control participants. Although, due to the retrospective nature of this study we did not have access to contact information on controls. Therefore, we assumed the out-of-pocket costs to be the same for controls as they are from the same region, with most of these costs related to travel. Additionally, deficient information regarding the long-term costs and health outcomes of pediatric patients undergoing Peds-TECH was considered a limitation of the current study. The QALY is a widely used generic measure for determining the allocative efficiency of health interventions. However, in this study, patient outcome measures such as QALYs were not available for cases and controls, which is also an important limitation for resource allocation decisions in the policy context. The limitation of not finding a statistically significant difference in the mortality could also be addressed by having a larger sample size in future studies of TM in pediatric population. Lastly, pre-intervention period data were not available. Therefore, pre-and-post comparisons for the trajectory of care and health outcomes between two time periods were not performed.

## Conclusions

Peds-TECH appears to be a cost-effective intervention for the care of emergency pediatric patients in community hospital ED settings. Furthermore, qualitative findings highlighted key OOP costs surrounding the use of the Peds-TECH program. Future research is warranted to assess the long-term costs and health outcomes of pediatric patients undergoing the Peds-TECH intervention on a larger provincial or national level. Our study findings have important implications as the economic evaluation is a core decision making factor in a resource constrained health system. A future provincial or national large-scale randomized control trial is needed to test the hypotheses using real-time data and to overcome challenges of sample size and establish causality. Furthermore, the qualitative findings from the interviews could be used to design a survey instrument for estimating the OOP costs and time/productivity losses for pediatric patients across the region.

## Data Availability

The datasets presented in this article are not readily available because this is personal hospital data which can not be shared. Requests to access the datasets should be directed to akhowaja@brocku.ca.
